# MicroRNA-155-5p Contributes to 5-Fluorouracil Resistance Through Down-Regulating TP53INP1 in Oral Squamous Cell Carcinoma

**DOI:** 10.3389/fonc.2021.706095

**Published:** 2022-01-06

**Authors:** Bowen Liu, Jingchao Hu, Han Zhao, Li Zhao, Shiyuan Pan

**Affiliations:** ^1^ Outpatient Department of Oral and Maxillofacial Surgery, Beijing Stomatological Hospital, School of Stomatology, Capital Medical University, Beijing, China; ^2^ Department of Periodontics, Beijing Stomatological Hospital, School of Stomatology, Capital Medical University, Beijing, China; ^3^ Multi-disciplinary Treatment Center, Beijing Stomatological Hospital, School of Stomatology, Capital Medical University, Beijing, China; ^4^ Department of Prosthodontics, Stomatological Hospital of Chongqing Medical University, Chongqing, China; ^5^ Chongqing Key Laboratory of Oral Diseases and Biomedical Sciences, Chongqing, China; ^6^ Chongqing Municipal Key Laboratory of Oral Biomedical Engineering of Higher Education, Chongqing, China; ^7^ Department of Dentistry, Chongqing Huamei Plastic Surgery Hospital, Chongqing, China

**Keywords:** microRNA-155-5p, 5-fluorouracil, resistance, TP53INP1, oral squamous cell carcinoma

## Abstract

The anticancer drug 5-fluorouracil (5-FU) resistance is a major obstacle to reducing the effectiveness of cancer treatment, and its detailed mechanism has not been fully elucidated. Here, in 5-FU-resistant human oral squamous cell carcinoma (OSCC) HSC3 cells (HSC3/5-FU), the levels of 21 miRNA candidates were detected using RT-PCR and miR-155-5p level increased strikingly in HSC3/5-FU cells compared to HSC3 cells. Compared with HSC3 cells, the CCK-8 assay showed that the HSC3/5-FU cells transfected with miR-155-5p inhibitors decreased 5-FU IC50. Ectopic expression of miR-155-5p in HSC3 and HSC4 cells increased 5-FU IC50 (CCK-8 assay), migration (wound-healing and transwell assays) and invasion (transwell assay) abilities. Seven miR-155-5p target candidates were discovered by miRNA prediction algorithms (miRDB, Targetscan, and miRWalk), and the RT-PCR results showed that in HSC3/5-FU cells TP53INP1 was of the lowest mRNA expression level compared with HSC3 cells. The RT-PCR and Western blotting assays showed that ectopic expression of miR-155-5p in HSC3 and HSC4 cells decreased TP53INP1 expression level. Furthermore, the luciferase reporter and RNA pull-down assays determined the interference effect of miR-155-5p on TP53INP1 expression. The enhancement of cell viability (CCK-8 assay), migration (wound-healing and transwell assays) and invasion (transwell assay) by miR-155-5p after 5-FU treatment was reversed by TP53INP1 overexpression. After treatment with 5-FU, HSC3-miR-155-5p tumor-bearing nude mice presented growing tumors, while HSC3-TP53INP1 group possessed shrinking tumors. In conclusion, these results lead to the proposal that miR-155-5p enhances 5-FU resistance by decreasing TP53INP1 expression in OSCC.

## Introduction

Globally, head and neck cancer is ranked as the sixth most common cancer and the majority cases are attributed to oral squamous cell carcinoma(OSCC) (1). Presently, a multidisciplinary approach, including surgery, radiotherapy, chemotherapy, or the combinations of these treatments has been applied for the treatment of OSCC patients (2). Although the treatment is effective for patients at the early stage, the 5-year survival rate of OSCC patients in advanced-stage remains <50% (3–5). Among the chemotherapy, 5-fluorouracil (5-FU) is commonly used and its active metabolites interferes with DNA and RNA synthesis, resulting to cell cycle arrest or cell death (6). However, the acquisition of resistance to 5-FU based chemotherapy leads to treatment failure in advanced and recurrent OSCC, resulting in a poor prognosis (7). Therefore, discovering the mechanism underlying the resistance to 5-FU of OSCC is of great importance for improving treatment regimen and bettering prognosis.

microRNAs (miRNAs/miRs) are critical gene expression regulators that bind to the 3’-untranslated region (3’-UTR) of target mRNAs, inducing mRNA degradation or translational suppression (8). Accumulating evidence has shown that aberrant expressions of miRNAs are involved in cancer progression by influencing various cell biological behaviors (9–11). miR-155 is one of the most important microRNAs, its upregulation increases cell growth, invasion, migration, stemness, and angiogenesis in many cancers (12). In OSCC, the overexpression of miR-155 facilitates proliferation and invasion of cancer cells and is associated with poor prognosis (13, 14). In addition, research documented that overexpressed miR-155 is found to be responsible for multidrug resistance in various tumor types (15–17). Notably, miRNA is associated with 5-FU resistance in colorectal cancer (17, 18). However, the role of miR-155 in 5-FU resistance of OSCC cells are still elusive.

Here, we displayed that miR-155 was increased in 5-FU resistant OSCC HSC3 cells. In silico analysis and functional studies further revealed that miR-155 contributes to 5-FU resistance in OSCC cells by targeting tumor protein p53 inducible nuclear protein 1 (TP53INP1). Our study suggests that miR-155 is a potential therapeutic target for overcoming 5-FU resistance during the treatment of OSCC.

## Results

### Establishment of miR-155-5p Associated with 5-FU Resistance in HSC3 Cells

**Figure 1:**
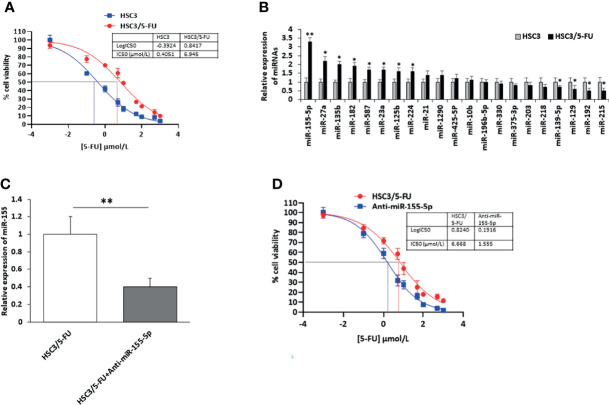
Upregulation of miR-155-5p in 5-FU resistant HSC3/5-FU cells. **(A)** The IC50 of 5-Fu in 5-FU resistant HSC3/5-FU cells. **(B)** The expression profile of miRNA candidates in HSC3/5-FU cells analyzed using RT-PCR. **(C)** The level of miR-155-5p in HSC3/5-FU cells with miR-155-5p inhibitors (Anti-miR-155-5p) was measured by RT-PCR. **(D)** The IC50 of 5-FU in HSC3/5-FU cells transfected with anti-miR-155-5p was measured using CCK-8 assay. IC50, half-maximal inhibitory concentration. *p < 0.05, **p < 0.01.

To identify miRNAs contributing to 5-FU resistance of OSCC cells, we first incubated HSC3 cells with 5-FU (HSC3/5-FU) and increased the concentration of 5-FU every two weeks. After 5 months, the IC 50 of 5-Fu in HSC3/5-FU cells were approximately 6.945 μM, it was estimated to be approximately seventeen folders higher than that to HSC3 cells (**Figure 1A**). Then, we further investigated the expression profile of miRNA candidates [miR-155 (18, 19), miR-27a (19), miR-135b (20), miR-182 (20), miR-587 (21), miR-23a (22), miR-125b (23), miR-224 (24), miR-21 (25), miR-1290 (26), miR-425-5P (27), miR-10b (28), miR-196b-5p (29),miR-330 (30), miR-375-3p (31), miR-203 (32), miR-218 (33), miR-139-5p (34), miR-129 (35), miR-192 (36), miR-215 (36)] in HSC3/5-FU and HSC3 cells and analyzed the expression of miRNAs using RT-PCR. The results showed that the expression of miR-155-5p was strikingly upregulated in HSC3/5-FU cells compared to HSC3 cells (**Figure 1B**). These results indicate that the deregulation of miR-155 might be involved in the 5-FU-resistance of OSCC cells.

To further investigate the role of miR-155-5p in the resistance to 5-FU in OSCC cells, we transfected HSC3/5-FU cells with negative control (NC) and miR-155-5p inhibitors (anti-miR-155-5p) and determined the expression level of miR-155-5p by RT-PCR (**Figure 1C**). As a result, half-maximal inhibitory concentration (IC50) was determined to be 1.555 μmol/L for Anti-miR-155-5p group (**Figure 1D**).

### MiR-155-5p Increased 5-FU Resistance in HSC3 and HSC4 Cells

**Figure 2:**
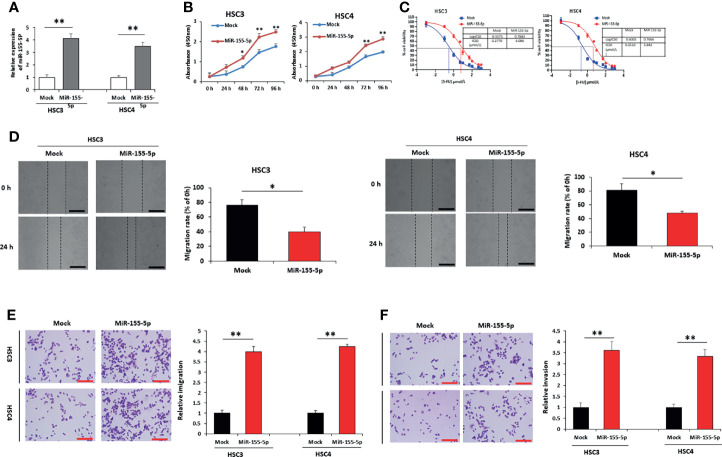
Over-expression of miR-155-5p induces resistance to 5-FU in HSC3 and HSC4 cells. **(A)** RT-PCR results showed the expression of miR-155-5p in HSC3 and HSC4 cells overexpressing miR-155-5p. **(B)** CCK-8 assay presented the proliferation of HSC3 and HSC4 cells overexpressing miR-155-5p. **(C)** The IC50 of 5-FU in HSC3 and HSC4 cells overexpressing miR-155-5p. Analysis of 5-FU-treated HSC3 and HSC4 cells transduction of miR-155-5p and mock vector using **(D)** the wound-healing assay, **(E)** the transwell migration assay, and **(F)** the transwell invasion assay. Scale bar, 100 μm. *p < 0.05, **p < 0.01.

To explore the influence of miR-155-5p on 5-FU resistance in OSCC cells, we transfected HSC3 and HSC4 cells with miR-155-5p mimic vector to overexpress miR-155-5p (**Figure 2A**), and the miR-155-5p increased cell viability (**Figure 2B**). IC50 was determined to be 6.086 μmol/L and 5.842 for the miR-155-5p overexpression HSC3 and HSC4 cells, while 0.277 μmol/L and 0.251 μmol/L for the HSC3 and HSC4 mock cells respectively (**Figure 2C**). The wound-healing assay (**Figure 2D**), transwell migration assay (**Figure 2E**) and transwell invasion assay (**Figure 2F**) showed that miR-155-5p increased sensitivity to 5-FU.

### MiR-155-5p Reduced TP53INP1 Expression in OSCC Cells

**Figure 3:**
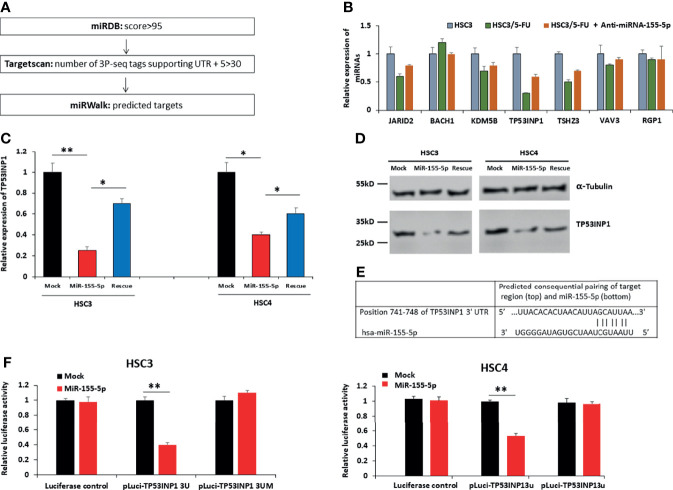
TP53INP1 is a direct target of miR-155-5p in OSCC cells. **(A)** Three different miRNA prediction algorithms (miRDB, Targetscan, and miRWalk) revealed miR-155-5p targets candidates. **(B)** The mRNA expression levels of miR-155-5p targets candidates in HSC3, HSC3/5-FU, HSC3/5-FU+Anti-miR-155-5p cells. **(C)** The mRNA and **(D)** protein levels of TP53INP1 in HSC3 and HSC4 cells infected with vectors expressing miR-155-5p mimic or miR-155-5p mimic +anti-miR-155-5p (rescue). **(E)** The prediction of the binding between miR-155-5p and TP53INP1 by Pictar. **(F)** Relative luciferase activity of the indicated *TP53INP1 reporter constructs* in HSC3 cells. *p < 0.05, **p < 0.01.

To discover the target mRNAs of miR-155-5p, we employed three different miRNA prediction algorithms (miRDB, Targetscan, and miRWalk) (37–40). A survey revealed that seven mRNAs, JARID2, BACH1, KDM5B, TP53INP1, TSHZ3, VAV3 and RGP1 are possible targets of miR-155-5p (**Figure 3A**, **Supplementary Table 2**), and we measured their mRNA expression levels in HSC3, HSC3/5-FU, HSC3/5-FU+Anti-miR-155-5p cells. The results showed that among the seven genes, TP53INP1 presented the lowest expression level in HSC3/5-FU clone, compared to HSC3 cells (**Figure 3B**). To assess the roles of miR-155-5p in TP53INP1 regulation, we analyzed both mRNA and protein levels of TP53INP1 after transfection of mock, miR-155-5p mimic, miR-155-5p mimic+Anti-miR-155-5p (Rescue) vector into HSC3 and HSC4 cells. The results showed that both mRNA and protein levels of TP53INP1 were downregulated by miR-155-5p (**Figures 3C, D**). The 3′-UTR of TP53INP1 mRNA contains a complementary site for the seed region of miR-155-5p (**Figure 3E**). To further confirm TP53INP1 regulation by miR-155-5p, we generated luciferase reporter constructs containing the TP53INP1 mRNA 3′UTR (741-748 nt) (pluci-TP53INP1 3U) or mutant 3′UTR missing the seed region binding sites for miR-155-5p (pluci-TP53INP1 3UM), and 48 h post-transfection, analyzed luciferase reporter expression after miR-155-5p over-expression. As shown in **Figure 3F**, miR-155-5p downregulated pluci-TP53INP1 3U reporter expression but did not affect the luciferase control or pluci-TP53INP1 3UM. Moreover, the RNA pull-down assay showed that TP53INP1 was enriched by biotinylated miR-155-5p, proving their direct interaction (**Supplementary Figure 2**). These results indicate that miR-155-5p negatively regulated the expression of TP53INP1 by binding to its 3′-UTR.

### MiR-155-5p Stimulates 5-FU Resistance by Targeting TP53INP1 in OSCC

**Figure 4:**
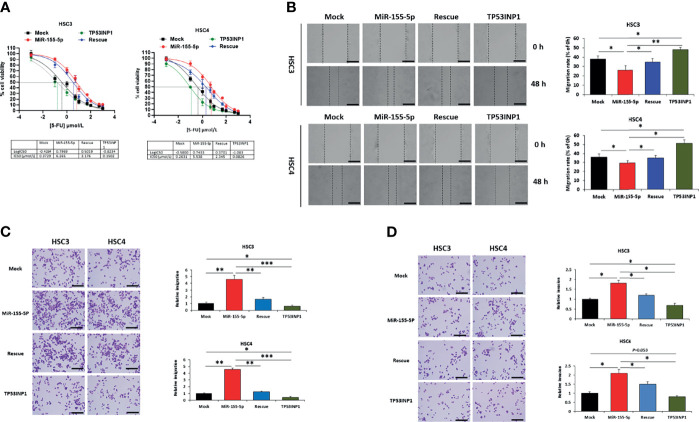
MiR-155-5p enhanced 5-FU resistance through targeting TP53INP1 in OSCC. After 5-FU treatment, **(A)** IC50 of 5-FU, **(B)** the wound-healing assay, **(C)** the transwell migration assay, and **(D)** the transwell invasion assay in HSC3 and HSC4 cells transfected with mock, miR-155-5p mimic, miR-155-5p+TP53INP1 (rescue) or TP53INP1 over-expression plasmid. Scale bar, 100 μm. *p < 0.05, **p < 0.01, *** *p* < 0.001.

We noticed that miR-155-5p increased cell viability after 5-FU treatment (**Figure 2B**) and downregulated TP53INP1 expression (**Figures 3C, D, F**). To further determine whether the increase in 5-FU resistance by miR-155-5p is mediated by the downregulation of TP53INP1, we investigated the sensitivity of HSC3-miR-155-5p and HSC4-miR-155-5p cells to 5-FU after over-expression of TP53INP1 (**Supplementary Figure 1**). As a result, increased cell viability (**Figure 4A**), migration (**Figures 4B, C**) and invasion (**Figure 4D**) by miR-155-5p after 5-FU treatment was reversed by over-expression of TP53INP1. These results demonstrated that the miR-155-5p regulated the 5-FU resistance of OSCC cells *via* targeting TP53INP1.

### MiR-155-5p Enhanced 5-FU Resistance of OSCC *In Vivo* by Targeting TP53INP1

**Figure 5:**
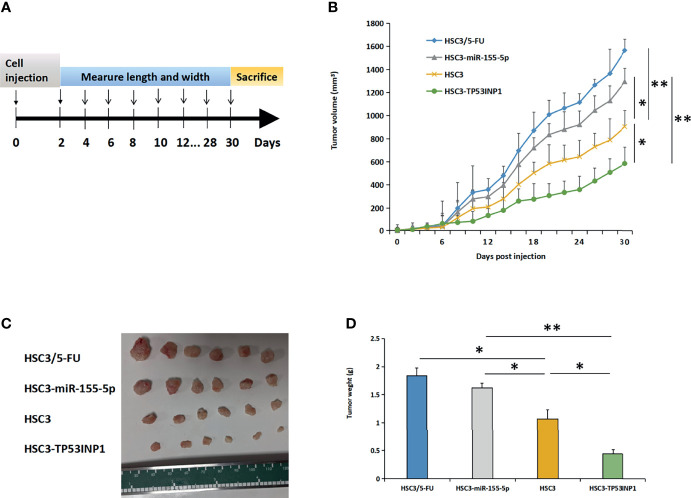
MiR-155-5p enhanced 5-FU resistance in OSCC mouse models by targeting TP53INP1. **(A)** HSC3, HSC3/5-FU, HSC3-miR-155-5p, or HSC3-TP53INP1 cells were subcutaneously injected into male athymic nude mice and were then treated with 5-FU. **(B)** The length and width of tumor bulks was measured every other day, tumor volume=length × width2 × π/6. Tumors were taken out, **(C)** photoed and **(D)** weighed. *p < 0.05, **p < 0.01.

To further explore the role of TP53INP1 in the treatment of OSCC using 5-FU, we constructed OSCC nude mice through subcutaneous injection of HSC3, HSC3/5-FU, HSC3-miR-155-5p, and TP53INP1 overexpression HSC3 (HSC3-TP53INP1) cells (**Figure 5A**). Then, we treated the OSCC nude mice with 5-FU and found that HSC3-TP53INP1 group possessed shrinking tumors compared with HSC3/5-FU and HSC3-miR-155-5p tumor-bearing mice (**Figures 5B–D**).

## Discussion

There are more than 650,000 oral cancer cases annually and OSCC accounts for >90% (41). Despite the current treatment is effective for patients at early stage, OSCC still has a high recurrence rate and causes 330,000 deaths every year (41, 42). As adjuvant chemotherapy, 5-FU is frequently used alongside surgery in patients with advanced OSCC. However, drug resistance makes tumor regression extremely difficult. And the mechanisms of the acquisition of drug resistance to 5-FU in OSCC cells are not fully understood. In this study, we constructed the 5-FU resistant cell HSC3/5-FU and explored the mechanism of miR-155-5p in the acquisition of 5-FU resistance in OSCC.

After inhibiting miR-155-5p in HSC3/5-FU cells, we found that the cell viability of 5-FU treated cells was reduced compared to the parental cells. We overexpressed miR-155-5p in HSC3 cell and processed it with 5-FU, miR-155-5p decreased sensitivity to 5-FU. In this study, we only selected the 5-FU resistant cell HSC3/5-FU, the selection results from this single cell line may not be common in OSCC. To explore whether this mechanism is universal in OSCC resistance to 5-FU, we also conducted the above-mentioned experiments in the OSCC cell line HSC4 and found consistent results. In addition to inhibiting tumor cell proliferation, it is also documented that 5-FU could suppress inhibit the migration and invasion of a variety of tumor cells (43–45). Consistent with these published researches, we found that 5-FU could also inhibit the migration and invasion of OSCC, and further experiments proved that miR-155-5p could attenuate these effects of 5-FU.

Because miRNAs function through interference with gene expression, we sought the target gene candidates of miR-155-5p using in silico analysis. TP53INP1 presented the lowest mRNA expression among the candidates in HSC3/5-FU cells, and this low expression could be improved by inhibiting miR-155-5p. MiR-155-5p overexpression suppressed the expression of TP53INP1 in both HSC3 while the rescue experiments could weaken this inhibition. Through luciferase assay and RNA pull-down, we determined the direct interference effect of miR-155-5p on TP53INP1.

Therefore, we focused on TP53INP1 for further exploration. This study showed that the TP53INP1 expression in OSCC is consistent with former research that it is often lost during cancer development from different organs (46–48). It has been documented that TP53INP1 exerts tumor suppressor function through involvement in cell death, cell-cycle arrest and cellular migration (47–49). It was found that miR-155-5p could promote epithelial-to-mesenchymal transition (EMT) through regulating TP53INP1 in paclitaxel-resistant gastric cancer cells (50). And exosomal miR-155-5p-5p could also enhance proliferation and migration capabilities of cancer cells by inhibiting TP53INP1 expression in gastric cancer (51). In addition, miR-155-5p could also promote liver cancer stem cell acquisition and self-renewal by targeting the gene TP53INP1 (52).

Up to now, there are few studies on the influence of miRNA on chemotherapy resistance through TP53INP1. It was reported that miR-182 increases cisplatin resistance in hepatocellular carcinoma cells by targeting TP53INP1 (53). Tie et al. claimed that let-7f-5p promotes 5-FU resistance and it could directly repress several pro-apoptotic proteins including TP53INP1 in colorectal cancer, indicating that TP53INP1 might negatively regulate 5-FU resistance (54). However, the regulation of TP53INP1 on 5-FU resistance still lacks direct evidence. In this study we demonstrated that miR-155-5p contributes to 5-FU through directly targeting the 3’ UTR of TP53INP1 in OSCC.

Because 5-FU could inhibit cell proliferation, migration, and invasion (55, 56), we evaluated the influence of miR-155-5p/TP53INP1 pathway on these capacities of HSC3/5-FU cells using the corresponding experiments. This study found that miR-155-5p suppressed the OSCC cell proliferation, migration and invasion through interfering with TP53INP1. Our findings confirmed that *in vivo*, resistance of OSCC to 5-FU was associated with the overexpression of miR-155-5p. In mice OSCC model accepting 5-FU treatment, HSC3-miR-155-5p cells-based tumor progressed rapidly, compared to that of the OSCC model based on HSC3 parent cells. Meanwhile, we demonstrated the role of TP53INP1 in improving the sensitivity of OSCC cells to 5-FU treatment. HSC3-TP53INP1 cells-based tumors were sensitive to 5-FU treatment, the tumor grew slowly, and has a good prognosis.

Our research provides novel insights into the role of miR-155-5p in the resistance of OSCC to 5-FU, which is conducive to the development of the use of miR-155-5p as a prognostic marker, or the use of anti-miR-155-5p as a treatment strategy to improve the efficacy of chemotherapy, especially 5-FU.

## Materials and Methods

### Cell Lines and Cell Culture

HSC3 and HSC4 cells were maintained in Dulbecco’s modified Eagle’s medium (DMEM, Invitrogen, Carlsbad, CA, USA) containing 10% fetal bovine serum (FBS) and 1% penicillin/streptomycin. The cells were cultured at 37°C in humidified air with 5% CO2.

### Establishment of 5-FU-Resistant Clones

To identify 5-FU resistant cell lines, human OSCC HSC3 cells were seeded in 10 cm dishes and first incubated with medium containing 0.2 μM 5-FU (Catalog No.: 51-21-8, Sigma-Aldrich, Shanghai, China). The 5-FU concentration increased 0.5 μM every two weeks. The selection continued for 5 months, and the final IC 50 of 5-Fu in HSC3/5-FU cells was 6.945 μM.

### *In Vitro* Cytotoxicity Tests

HCS3 or HSC4 cells were plated in triplicate at 1 × 10^4^ cells per well in 96-well plates. Four hours later, 5-FU in different concentrations was added and incubated for 72 h.

### Cell Viability Assay

Cell viability was assessed using CCK-8 assay (Catalog No.: 96992, Sigma-Aldrich), and it was conducted following manufacture’ instruction (https://www.sigmaaldrich.cn/CN/en/product/sigma/96992?context=product). In brief, cells in 24-well plates were transfected with miR-155-5p mimic, inhibitor or control miRNA and incubated with 5-FU for 72 h and then further incubated with CCK-8 for 4 h. CCK-8 assay was performed at 0, 24, 48, 72, 96 h after seeding cells. All experiments were performed at least three times.

### Cell Wound-Healing Assay

Cell migration was assessed using wound-healing assay. HSC3 or HSC4 cells transfected with miR-155 grew to confluence in 24-well plates, cells were scratched using a sterile 200 μL pipette tip and maintained in serum-free medium. Images were taken at 0, 24, 48h. Wound closure was photographed along the scrape line using phase contrast microscopy (Nikon Eclipse TS100 inverted microscope). The wound distance was calculated based on the distance migrated compared to the original scratch width.

### Transwell Assay

The chambers were washed thoroughly with 10 mM PBS, fixed in 4% paraformaldehyde for 30 min, and stained with 0.2% crystal violet for 10 min. Non-invading cells, from the membrane upper surface, were removed using a cotton swab. The membranes containing the invaded cells (under the surface of membrane), were photographed. Images of three random microscope fields were captured in duplicate, using an inverted optical microscope (Floid Cell Imaging Station, Life Technologies, Carlsbad, CA, USA). The areas of cell invasion were determined by Image J software.

Cell migration and invasion were analyzed using transwell culture system. To detect cell migration, 2×10^5^ cells in 200μl serum-free DMEM were seeded into the upper chambers, and DMEM containing 20% FBS was added to the lower chamber as a chemoattractant. Following incubation at 37°C for 24 h, supernatant was discarded and cells in the membrane upper surface were removed using a cotton swab. Invasive cells located on the lower surface were fixed with paraformaldehyde (Thermo Fisher Scientific) and stained with 0.1% crystal violet (Catalog No.: V5265, Sigma-Aldrich) for 10 min at room temperature. To detect cell invasion, the inner sides of the Transwell chambers were pre-coated with Matrigel (BD Biosciences, San Jose, CA, USA) at room temperature, after which, the procedure was performed to assess cell invasion. An inverted optical microscope (Floid Cell Imaging Station, Life Technologies, Carlsbad, CA, USA) was used to count and photograph the migrated and invaded cells. The areas of cell migration and invasion were determined by Image J software (NIH, Bethesda, MD, USA).

### Luciferase Reporter Assay

To confirm the binding of miR-155a-5p to TP53INP1 mRNA, a Dual-Luciferase Reporter Assay System (cat. no. E1910; Promega Corporation) was utilized according to the manufacturer’s instructions. Briefly, miR-155a-5p mimics (5’-UUACACACUAACAUUAGCAUUAA-3’) and a miR-155a-5p mutant (5’-UUACACACUAACAUUGAAUCGUA-3’) were synthesized by BioSunne Inc. (Shanghai, China). The Luciferase control, pLuci-TP53INP1 3U or pLuci-TP53INP1 3UM luciferase reporter plasmid (8 µg) and NC (GFP plasmid) or miR-155a-5p mimic plasmid (100 ppm) were transfected into HSC3 cells using Lipofectamine 3000 Transfection Reagent (Catalog No.: L3000015, Thermo Fisher Scientific.). At 48 h following transfection, the cells were lysed, and the luciferase activity was measured using the Reporter Assay System (Promega Corporation). Cotransfection experiments were carried out in triplicate.

### RNA Pull-Down

The biotin-labeled miR-155-5p mimic, biotin-labeled mutated miR-155-5p and negative control were transfected into HSC3 and HSC4 cells. After 48 h, the cells were harvested and lysed, and the lysate was added to the Dynabeads™ M-280 streptavidin magnetic beads (Invitrogen, Carlsbad, CA, USA). The mixture was incubated at room temperature for 15-30 min. The enrichment of the co-deposited TP53INP1 RNA was determined using RT-PCR.

### Real-Time Reverse Transcription PCR

The expression levels of miRNA and miR-155a-5p target candidate mRNA were analyzed *via* the real-time reverse transcription PCR (RT-PCR). Total RNAs were isolated for mRNA analysis before cDNAs were prepared *via* reverse transcription, The abundance of transcripts was assessed by RT-PCR analysis using LightCycler^®^ RNA Master SYBR Green I (Roche, Basel, Switzerland) and gene-specific primer sets on a StepOne Plus instrument (Applied Biosystems, Foster City, CA, United States). The relative level of miRNA or mRNA was normalized to the U6 RNA or GAPDH mRNA using the comparative delta CT (2-ΔΔCT) method. Primer sequences are listed in **Supplementary Tables 1** and **3**.

### Western Blotting

Whole cell lysates were prepared using RIPA buffer (Catalog No.: 89900, Thermo Fisher Scientific, Waltham, MA, USA), and the protein concentration was measured using Pierce™ BCA Protein Assay Kit (Catalog No.: 23227, Thermo Fisher Scientific). Protein extracts were separated by electrophoresis in SDS-containing polyacrylamide gels, and then transferred onto Immun-Blot^®^ PVDF Membrane (Catalog No.: 162017, BIO-RAD, Hercules, CA, USA). The membranes were incubated with the primary rabbit monoclonal antibody against human TP53INP1 (Catalog No.: MA5-34751, Thermo Fisher Scientific) or α-tublin (Catalog No.: A11126, Thermo Fisher Scientific) and then incubated with HRP-conjugated goat anti-Rabbit IgG secondary antibody conjugated to horseradish peroxidase (Catalog No.: 31466, Thermo Fisher Scientific). GAPDH was used as an internal control.

### Animal Experiments

To evaluate the roles of miR-155-5p and TP53INP1 in 5-FU resistance in OSCC, we injected HSC3, HSC3/5-FU, HSC3-miR-155-5p, or HSC3-TP53INP1 cells (1×10^7^ cells/100 μl DMEM/mouse) subcutaneously into male athymic nude mice (6-week-old). All mice were treated with 5-FU in the concentration of 6.945 μmol/L. The volume of tumor bulks was measured per 2 days using digital calipers and were calculated using the formula: volume= length × width2 × π/6. 1 month after cell inoculation, all mice were sacrificed, and tumors were taken out and weighed. The animal studies were approved by Animal Welfare and Research Ethics Committee of Capital Medical University, and the animal experiments were conducted in accordance with the institutional and national regulations.

### Statistics

All data are expressed as mean ± standard deviation. Statistical analysis was performed using GraphPad Prism 8.0 (GraphPad Software, CA, USA). Student’s t-test (two-tailed) was used to compare two groups, and analysis of variance was used to compare multiple groups. The differences were considered statistically significant when p < 0.05, and indicated as * (P < 0.05), ** (0.05< P < 0.001), and *** (P < 0.001). All experiments were performed in triplicate.

## Data Availability Statement

The original contributions presented in the study are included in the article/**Supplementary Material**. Further inquiries can be directed to the corresponding author.

## Ethics Statement

The animal study was reviewed and approved by Animal Welfare and Research Ethics Committee of Capital Medical University.

## Author Contributions

BL designed the research, conducted experiments, analyzed the data, wrote the manuscript, and replied to the reviewers. JH and HZ performed *in vitro* experiments and analyzed data. LZ designed the research design and revised the manuscript. SP replied to the reviewers, designed research, conducted experiments, analyzed data, and revised the final manuscript. All authors contributed to the article and approved the submitted version.

## Funding

This work was supported by Discipline Construction Fund from the Beijing Stomatological Hospital, School of Stomatology, Capital Medical University (17-09-09 to BL).

## Conflict of Interest

The authors declare that the research was conducted in the absence of any commercial or financial relationships that could be construed as a potential conflict of interest.

## Publisher’s Note

All claims expressed in this article are solely those of the authors and do not necessarily represent those of their affiliated organizations, or those of the publisher, the editors and the reviewers. Any product that may be evaluated in this article, or claim that may be made by its manufacturer, is not guaranteed or endorsed by the publisher.
