# Supplementary material for: MicroRNA-155-5p Contributes to 5-Fluorouracil Resistance Through Down-Regulating TP53INP1 in Oral Squamous Cell Carcinoma
Source: Front Oncol. 2022 Jan 6;11:706095. doi: 10.3389/fonc.2021.706095 (PMC8770267; doi:10.3389/fonc.2021.706095)
Supplement: Supplementary file 1 [file DataSheet_1.docx]

Supplementary Material

**Supplementary Table 1. Primer sequences of miRNA used for real-time RT-PCR.**

| microRNA | **Forward (5′→3′)** | **Reverse (5′→3′)** |
| --- | --- | --- |
| miR-155-5p | TAATGCTAATCGTGATAGGGGTTC | CTCAACTGGTGTCGTGGAGTC |
| miR-27a | GCGCTTCACAGTGGCTAAG | GTGCAGGGTCCGAGGT |
| miR-135b | GGTATGGCTTTTCATTCCT | CAGTGCGTGTCGTGGAGT |
| miR-182 | ATCACTTTTGGCAATGGTAGAACT | TATGGTTTTGACGACTGTGTGAT |
| miR-587 | CCAGGCAA-GAGAGAGTTGCTG | ATGG-GCTTTCCACTGGTGATG |
| miR-23a | GCGATCACATTGCCAGGG | AGTGCAGGGTCCGAGGTATT |
| miR-125b | TCCAGTTCGGGAAGTGAAGTGA | GTCCAAAGTGGTATTGCCACTA |
| miR-224 | GCTCAAGTCACTAGTGGTTCC | CAGTGCGTGTCGTGGAGT |
| miR-21 | TGGGCTTATCAGACTGATGTTGA | CTCAACTGGTGTCGTGGAGTC |
| miR-1290 | ACACTCCAGCTGGGTGGATTTTTGGATC | CTCAACTGGTGTC |
| miR-425-5P | TGCGGAATGACACGATCACTCCCG | CCAGTGCAGGGTCCGAGGT |
| miR-10b | TACCCTGTAGAACCGAATTTGTG | CAGTGCGTGTCGTGGAGT |
| miR-196b-5p | GCGCGTAGGTAGTTTCCTGTT | AGTGCAGGGTCCGAGGTATT |
| miR-330 | GACCCTTTGGCGATCTCTG | CTGTGCTTTGCTCGTTGGAT |
| miR-375-3p | CGGGTTTGTTCGTTCGGCT | GTGCAGGGTCCGAGGTATT |
| miR-203 | GTATTCGCACTGGATACGACCGACC | TGCGCTAACAGTCTACAGCCA |
| miR-218 | GCCGAGTTGTGCTTGATC | CTCAACTGGTGTCGTGGA |
| miR-139-5p | GCCTCTACAGTGCACGTGTCTC | CGCTGTTCTCATCTGTCTCGC |
| miR-129 | AAGCCCTTACCCCAAAAAGTAT | CTTTTTGCGGTCTGGGCTTGC |
| miR-192 | CTGACCTATGAATTGACAGCCGT | ATCCAGTGCAGGGTCCGA |
| miR-215 | CTCGAGATGTCATCCTCAG | GAATTCGTGAGTTCTTCTG |
| U6 | CTCGCTTCGGCAGCACAT | AACGCTTCACGAATTTGCGT |

**Supplementary Table 2.** **The screening of miR-155-5p target candidates using miRNA prediction algorithms.**

| **MiRNA prediction algorithms** | **Screening criteria** | **Genes** |
| --- | --- | --- |
| miRDB | Score>95 | JARID2、ZNF652、BACH1、ZIC3、IRF2BP2、KDM5B、ZNF98、OLFML3、TBR1、CDX1、CHAF1A、MPEG1、FOS、ACTL7A、MARCH1、IKBIP、TP53INP1、GYG1、MYBL1、GABARAPL1、KANSL1、TSHZ3、VAV3、RGP1 |
| Targetscan | Number of 3P-seq tags supporting UTR + 5>30 | [JARID2](http://www.ncbi.nlm.nih.gov/entrez/query.fcgi?db=gene&cmd=Retrieve&dopt=full_report&list_uids=3720), [BACH1](http://www.ncbi.nlm.nih.gov/entrez/query.fcgi?db=gene&cmd=Retrieve&dopt=full_report&list_uids=571), [IRF2BP2](http://www.ncbi.nlm.nih.gov/entrez/query.fcgi?db=gene&cmd=Retrieve&dopt=full_report&list_uids=359948), [KDM5B](http://www.ncbi.nlm.nih.gov/entrez/query.fcgi?db=gene&cmd=Retrieve&dopt=full_report&list_uids=10765), [CHAF1A](http://www.ncbi.nlm.nih.gov/entrez/query.fcgi?db=gene&cmd=Retrieve&dopt=full_report&list_uids=10036), [FOS](http://www.ncbi.nlm.nih.gov/entrez/query.fcgi?db=gene&cmd=Retrieve&dopt=full_report&list_uids=2353), TP53INP1, [TSHZ3](http://www.ncbi.nlm.nih.gov/entrez/query.fcgi?db=gene&cmd=Retrieve&dopt=full_report&list_uids=57616), [VAV3](http://www.ncbi.nlm.nih.gov/entrez/query.fcgi?db=gene&cmd=Retrieve&dopt=full_report&list_uids=10451), [RGP1](http://www.ncbi.nlm.nih.gov/entrez/query.fcgi?db=gene&cmd=Retrieve&dopt=full_report&list_uids=9827) |
| miRWalk | Predicted Targets | [JARID2](http://www.ncbi.nlm.nih.gov/entrez/query.fcgi?db=gene&cmd=Retrieve&dopt=full_report&list_uids=3720), [BACH1](http://www.ncbi.nlm.nih.gov/entrez/query.fcgi?db=gene&cmd=Retrieve&dopt=full_report&list_uids=571), [KDM5B](http://www.ncbi.nlm.nih.gov/entrez/query.fcgi?db=gene&cmd=Retrieve&dopt=full_report&list_uids=10765), TP53INP1, [TSHZ3](http://www.ncbi.nlm.nih.gov/entrez/query.fcgi?db=gene&cmd=Retrieve&dopt=full_report&list_uids=57616), [VAV3](http://www.ncbi.nlm.nih.gov/entrez/query.fcgi?db=gene&cmd=Retrieve&dopt=full_report&list_uids=10451), [RGP1](http://www.ncbi.nlm.nih.gov/entrez/query.fcgi?db=gene&cmd=Retrieve&dopt=full_report&list_uids=9827) |

**Supplementary Table 3. Primer sequences of miR-155-5p target candidates used for real-time RT-PCR.**

| **Gene** | **Forward (5′→3′)** | **Reverse (5′→3′)** | **Product length (bp)** |
| --- | --- | --- | --- |
| [JARID2](http://www.ncbi.nlm.nih.gov/entrez/query.fcgi?db=gene&cmd=Retrieve&dopt=full_report&list_uids=3720) | ACCAGTCTAAGGGATTAGGACC | TGCTGGGACTATTCGGCTGA | 183 |
| [BACH1](http://www.ncbi.nlm.nih.gov/entrez/query.fcgi?db=gene&cmd=Retrieve&dopt=full_report&list_uids=571) | CTCAGCCTTAATGACCAGCGG | GCCTACGATTCTTGAGTGGAAG | 132 |
| [KDM5B](http://www.ncbi.nlm.nih.gov/entrez/query.fcgi?db=gene&cmd=Retrieve&dopt=full_report&list_uids=10765) | CCATAGCCGAGCAGACTGG | GGATACGTGGCGTAAAATGAAGT | 102 |
| TP53INP1 | TTCCTCCAACCAAGAACCAGA | GCTCAGTAGGTGACTCTTCACT | 140 |
| [TSHZ3](http://www.ncbi.nlm.nih.gov/entrez/query.fcgi?db=gene&cmd=Retrieve&dopt=full_report&list_uids=57616) | GTGACCGAATGGCTGACTTTG | AGGCTATCCGACACAGTCGT | 100 |
| [VAV3](http://www.ncbi.nlm.nih.gov/entrez/query.fcgi?db=gene&cmd=Retrieve&dopt=full_report&list_uids=10451) | GCCGGGGATACCGTTGAAC | CAAGGCTTGACTGCATCACT | 113 |
| [RGP1](http://www.ncbi.nlm.nih.gov/entrez/query.fcgi?db=gene&cmd=Retrieve&dopt=full_report&list_uids=9827) | CTGTGACCTGAGGCTTGATCC | AGTGATAGGGGAGTTGACACG | 151 |
| GAPDH | TGTTCGTCATGGGTGTGAAC | ATGGCATGGACTGTGGTCAT | 131 |


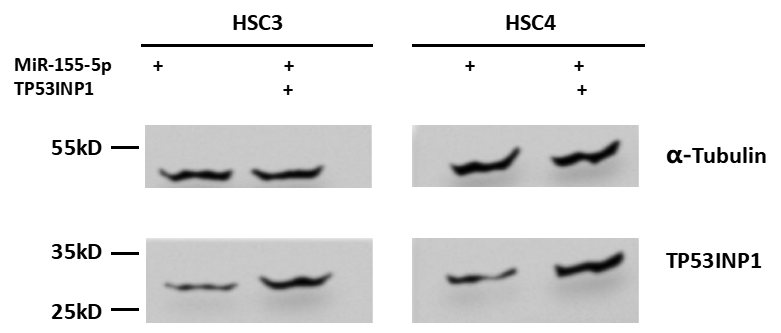


**Supplementary Figure 1. TP53INP1 protein level.** HSC3 and HSC4 cells transfected with miR-155-5p or miR-155-5p+TP53INP1 over-expression plasmid, and the TP53INP1 protein level was analysed using Wetern blotting assay.


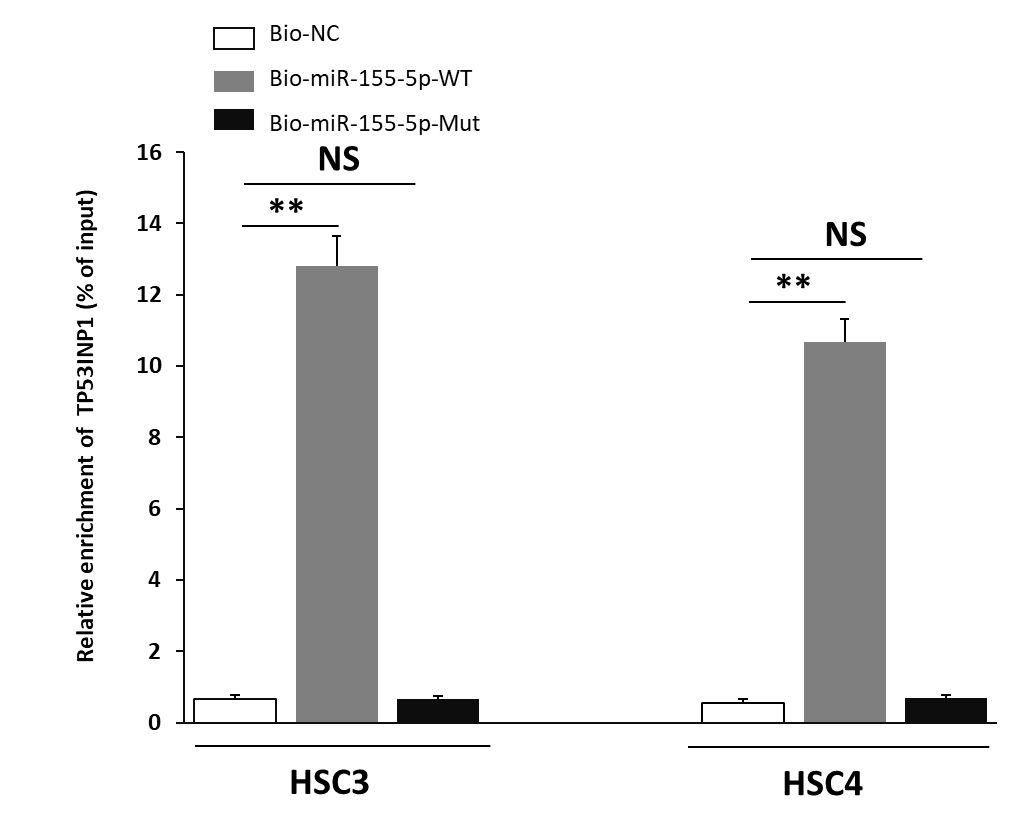


**Supplementary Figure 2. Verification of the relationship between miR-155-5p and TP53INP1 by RNA pull-down assay.** Bio-NC, biotin-labeled negative control. Bio-miR-155-5p-WT, biotin-labeled miR-155-5p mimic. Bio-miR-155-5p-Mut, biotin-labeled mutated miR-155-5p**.***, p<0.05; * *, p<0.01. Triple experiments were conducted.
